# Developing a Writing Workshop for Clinician-Educators: A Synergistic Integration of Ethics, Rhetoric and Education Theories, and Social Science

**DOI:** 10.15694/mep.2017.000137

**Published:** 2017-07-31

**Authors:** B. Lee Ligon, Rachelle Weinstein, Satid Thammasitboon

**Affiliations:** 1 Baylor College of Medicine; 2 Texas Children's Hospital; 3 Baylor College of Medicine

**Keywords:** ethics, education theory, rhetoric, writing workshop

## Abstract

This article was migrated. The article was marked as recommended.

**Introduction:** Clinician-educators often need specific direction in writing to publish their scholarly works. Faculty writing workshops and retreats, as well as helpful articles, address the “how-to” of writing but not the “pitfalls” of publishing. We describe an innovative, interactive workshop we crafted for the medical education profession, using our material from didactic presentations on writing given locally and internationally and incorporating education, rhetoric, and social science theories.

**Methods:** Using baseball as a metaphor, we present the material in three sections: “Know the Playing Field” (publication ethics, journal selection/timing, accountability, boundaries), “Learn New Strategies” (publication standards, rhetorical statement, IMRAD), and “Score the Home Run!” (finalizing and submitting the manuscript, responding to reviewers).

**Results**: It has been given in numerous venues, adapted to cover time constraints ranging from 1.5 hours to 3 hours, presented to audiences ranging from Executive Directors to medical trainees, and adjusted in response to feedback, demonstrating its flexibility. In every instance, it has been lauded for its educational value; participants have rated the overall quality of the workshop, as well as individual components, as good or excellent (all above 4 on 1-5 scale) and lessons learned as very useful and most useful.

**Conclusion:** We offer it as a practical and flexible framework for adaptation by other institutions.

## Introduction

Clinician-educators often are overwhelmed by the need to publish their scholarship, which for some is a daunting, even seemingly impossible, task ( Boice 1989; Keyes 1995; Steinert et al, 2008; Malani and Lypson 2012; Sullivan 2013), especially so for those who received little or no education in writing during their years of training ( Simpson et al 2000; Sullivan 2013; Singh and Mayer 2014). Further, despite the encouraging work being done to address local needs (e.g., creating curriculum, assessment), clinician-educators often have difficulty disseminating their products, which is required for recognized scholarship ( Bligh and Brice 2009; Simpson et al 2013). In our Department, the largest pediatric department in the nation, 70% of our faculty identified themselves as clinician-educators. The Chairman forged an education enterprise to recognize and empower the faculty to engage in scholarship in medical education ( Thammasitboon et al 2017). Although a major goal was to recognize the various forms of educational scholarship, in addition to publications, a component also was incorporated to provide training in writing, through the “hub” of the enterprise, the Center for Research, Innovation and Scholarship in Medical Education (CRIS). We offer several didactic presentations, and from them we have crafted an interactive workshop that incorporates educational and discourse theories, as well as psychosocial components. The literature attests to the value of providing faculty writing workshops and retreats for training in writing ( Steinert et al 2008; Boudreu et al 2012) and authors with writing experience have published helpful articles ( Audisio et al 2009; Sullivan 2012; Azer et al 2014; Ligon et al 2017) and shorter “tips” ( Hong 2014; Norman 2014; Cook 2016) on writing. Our targeted needs assessment was based on the literature, the first-hand experiences of the authors, and feedback from the didactic presentations. We describe herein important features of our workshops that render them unique in the approach to writing for the medical education profession, with the intention of providing a framework for other medical education writing programs.

## Methods

### Workshop Development

Three of the CRIS faculty members collaborated on creating a faculty development workshop based on a didactic scientific writing presentation that one of them (BLL) had been giving for years in local, national, and international medical/science venues. Through collective expertise, we transformed the didactic presentation into an interactive, hands-on scholarly writing workshop for clinician-educators. We first decided to use the didactic material that addressed numerous shortcomings in medical writing, exhibited by both clinicians and educators, that originally was presented as “Pitfalls to Avoid.” They include adhering to ethical standards, crafting a rhetorical statement, formulating a well-written manuscript (e.g., grammar, economy of words), following publisher’s instructions, dealing with peer reviewers, and resubmission of a manuscript. A separate didactic presentation focused on specific challenges of writing the scholarly medical/scientific manuscript. We restructured the didactic materials from these two separate but equally important presentations into a cohesive, three-part workshop that incorporated Kolb’s experiential learning theory for hands-on, interactive opportunities for the participants ( Kolb 1983; Kolb et al 2000; Kolb AY and Kolb DA 2008); Kinneavy’s theory on “aims of discourse” ( Kinneavy 1969);” and psychosocial implications regarding disruptions, boundaries, and time management. As the basic outline for science/medical manuscripts (IMRAD: Introduction, Materials/Methods, Results, Discussion/Conclusion) coincides with most medical education manuscripts, we easily converted the focus from clinical or basic research to medical education research and scholarship. To aid in the participants’ engagement, we designed and provided a workbook that includes the entire presentation in reduced form, links to vital information, copies of some important supporting articles, and templates that we designed for writing abstracts, manuscripts, and case reports.

### Workshop Components

The result is a three-part workshop that can be adjusted from a two-hour interactive seminar to a full-day writing retreat. We describe herein the basic workshop and ways to adjust the presentation dependent upon the focus, time constraints, and priorities. We structured the workshop around a sports metaphor, football, and named the three sections according to what one would expect when engaging in that sport: “Know the Playing Field,” “Learn New Strategies,” and “Score the Touchdown!”

#### “Know the Playing Field”

This portion of the presentation focuses on matters the author needs to know before starting to write, namely potential pitfalls, defined as “an unsuspected difficulty or danger,” “a hidden hazard,” or “an unforeseen or unexpected or surprising difficulty.” These pitfalls were grouped according to four overarching concerns: ethics, journal selection and timing of submission, accountability, and setting boundaries.

*Ethics.* The first topic that we address is publishing ethics, based on the international appreciation for the Committee on Publication Ethics (COPE ethics). This topic is very complex and comprehensive, requiring a session of its own to address it fully, so we focus on only two pitfalls, ones that we have encountered on numerous occasions and appear to be disregarded or even unknown in different quarters, even among senior faculty. The first is *questionable authorship.* We have the participants list the potential authors involved in the scholarly activity they plan to publish. We then explain the criteria for authorship: what constitutes authorship and what does *not* constitute authorship. Again, these criteria are based on COPE standards (COPE Authorship), which we outline in the presentation and workbook, along with the definition of the role of authors and contributors from the International Committee of Medical Journal Editors (ICMJE). Next, the participants review the list of potential authors and identify the ones who meet the criteri, after which we have a Q&A to address particular concerns. The second topic is one that generates considerable concern, as it can ruin a scholar’s reputation: *plagiarism.* Although most people encounter this issue early in their academic training, it continues to plague the academic world and has become rampant in some quarters, especially with the advent of the internet and easy access to volumes of material. However, its repercussions have not been diminished. We define what constitutes plagiarism (“What is..”), explain the concept of self-plagiarism, include a link to a “plagiarism quiz” (WriteCheck Quiz), describe some examples from our own editing experiences, and provide information on CrossCheck, a plagiarism-checking service that Cross Ref launched in 2008. The participants then engage in an interactive exercise in which we first demonstrate actual plagiarism in one set of samples, *marked* to indicate the plagiarism, and then provide time for participants to work on another set of *unmarked* paragraphs to identify plagiarism in that sample. In the workbook, we include published examples of the public exposure and humiliation individuals have experienced who committed plagiarism ( Chalmers 2006; Masha 2009).

*Journal Selection/Timing.* The second topic concerns “where” and “when” to submit a manuscript. The early identification of an appropriate journal obviates the need to change the format of the text and the references at a later date and directs the focus of the manuscript. With regard to “where,” we stress the importance of identifying the appropriate audience (e.g., *Pediatrics* is not likely to be a good option for a manuscript on nursing practices) and caution against two tendencies: to aim “too high,” as high-impact journals have extensive competition, or, contrariwise, to aim “too low,” as a journal that accepts almost anything will not give the clinician-educator the recognition that his/her scholarly work deserves. We suggest that the participants use JANE (JANE), SCImago Journal & Country Rank (SCImago), and/or Think.Check.Submit (ThinkCheckSubmit) to identify a proper journal. We also warn them against “predator” journals and direct them to a source for identifying such journals. We include in the workbook an article on chronicling predatory open access publishers ( Beall 2013). With regard to “when” the manuscript is ready to be released, we explain the dichotomies of a) rushing to publication before the manuscript is publishable and b) delaying so long that the information is published by someone else or obsolete.

*Accountability.* Having some form of accountability is critical to the writing process ( Steinert 2008). Some people are self-motivated and need only their own schedules, whereas other educators may need to have someone to whom they answer or give account of their progress in writing a manuscript. We address three hurdles we have witnessed individuals encounter: procrastination, group authorship, and individual responsibility. During this session, we provide an opportunity, first, for the participants to discuss among themselves why they procrastinate and then we ask for feedback. The typical responses that we address are: “I just don’t have time,” “I don’t feel inspired to write,” and “I simply can’t write; never could and still can’t.” We next address the sensitive area of identifying the order of authors in multi-author manuscripts, using Kolb’s experiential learning theory to guide an interactive activity ( Figure 1). This portion of the workshop has generated considerable engagement of the participants. The third aspect of accountability that we address is individual responsibility - set and keep deadlines!

**Figure:**
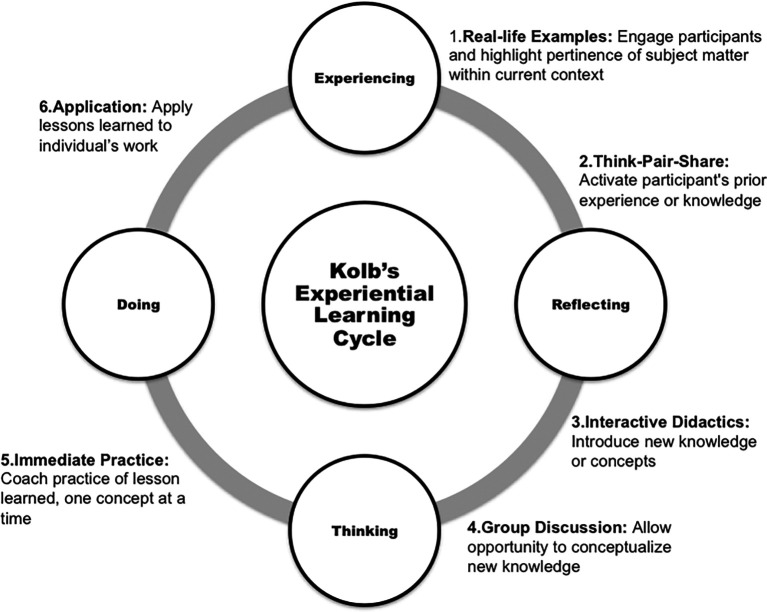


*Boundaries.* Setting boundaries (i.e., protecting oneself) ( Cloud 1992) is crucial to having a successful writing experience. This portion of the workshop looks at psychosocial components. The presenter discusses three topics: setting aside designated time, finding the right place, and dealing with distractions. For each of these self-preservation areas, we offer different suggestions that have worked for other people. We also provide some basic information on the psychological impact of being interrupted ( Adamczyk and Bailey 2004), especially by cell phones (ICMJE), and how to ward off interruptions.

#### “Learn New Strategies”

The second session deals with the manuscript itself. Just as a football player needs to constantly be learning new strategies, writers need to hone their skills. We provide several means to enhance their writing experience.

*Publication Standards.* We explain the importance of obtaining a journal’s instructions and the value of making a checklist *before* they get started and of reviewing it before submitting the manuscript. We also direct the participants to “The Uniform Requirements for Manuscripts,” providing the link in the workbook. [http://www.icmje.org/recommendations].

*Rhetorical Statement.* One of the authors with special training in rhetoric uses Kinneavy’s theory on aims of discourse ( Figure 2) and actual incidents to illustrate how a well-crafted rhetorical statement is essential to successful scholarly communication. The didactic portion explains that simply reporting their scholarly work is not sufficient: they must state succinctly *why* it is important and what it offers to the medical education community. For the interactive exercise, we provide time for participants to think through questions we pose (e.g., “What relevance does my scholarly work have?”), to write out succinct statements describing the value of their projects, and to share their statements in small groups to receive feedback from their peers. If time allows, each group may read one of the rhetorical statements and have the entire audience respond with questions and/or suggestions.

**Figure:**
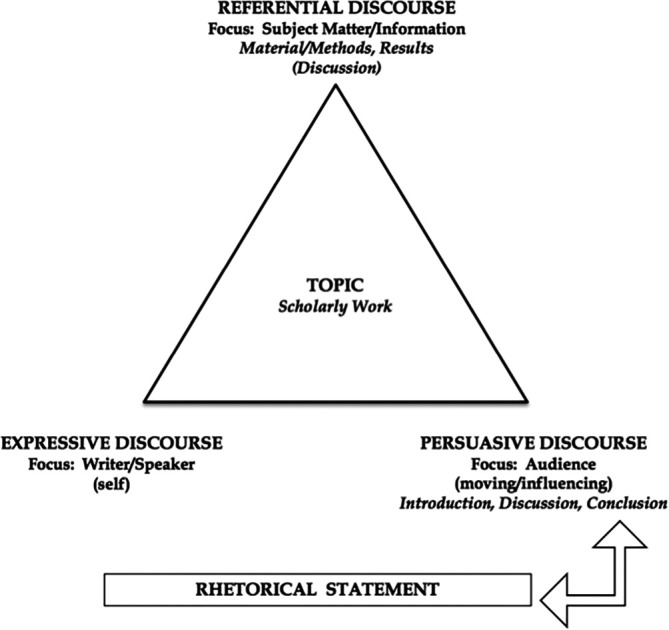


*Manuscript Format.* During this session, we concentrate primarily on what *does* and what *doesnot* go into each section of the “IMRAD” - introduction, materials, results, discussion - format. We explain the purpose of each section (e.g., *Introduction* provides the context and captures the reader’s attention) and emphasize the important difference between the *Materials/Methods*, likening it to a recipe ( *what* you did and *how* you did it- remember, the purpose being to allow for replication), and *Results* (what happened). We stress that all other information needs to be saved for the *Discussion.* After the didactic presentation on the *Introduction*, we allow time for the participants to write an introductory sentence, supply bullet-point ideas for background, and insert the already crafted rhetorical statement as the last sentence, using the template we provide. For longer sessions, participants write a full introduction for a project they have finished or that they expect to complete soon. We follow a similar format for the *Material/Methods* section. We provide a section in the template for the results but offer that portion as a take-home, as some of them may not have completed their desired project. We then explain how to provide in the *Discussion* the “why” and “wherefore,” along with comparisons of results with other studies or scholarly findings. We also reiterate the importance of stating the significance of the scholarly work (i.e., a rewording of the rhetorical statement). The *Conclusion* should explain any drawbacks, state any cautions, and provide an interpretation of what the results mean going forward. We close this portion with a short statement on the *Acknowledgements.*

#### “Score the Home Run!”

The last session deals with fine-tuning the manuscript, submitting it, and responding to reviewers.

*Prepare the Manuscript for Submission:Make Revisions.* We explain the writing *process*, namely the need for rewriting..and rewriting..and rewriting. *Proofread.* We emphasize the value of proofing the text and of getting someone else to offer a new set of “eyes.” *Check the Punctuation.* We use this time for some humor, as everyone is tired by this point. We give humorous examples of how the use of punctuation can completely change the meaning of a sentence or paragraph. *Check the Prose.* We explain how to eliminate excessive verbiage and avoid hedging *. Check Figures/Tables.* Data in tables/figures must match the text. Discrepancies can have negative implications, so we offer some examples that we have encountered of potential disasters averted by careful proofing.

*Submit the Manuscript.* The time has come to submit the manuscript and to wait while it goes through the peer-review process. This waiting can be emotionally draining, as one has put considerable time and effort into crafting the product. We encourage them to be patient and to concentrate on other projects, as the “ball is now in the reviewers’ court.”

*Respond to the Reviewers.* What happens if you get “Rejected”? Every author knows how distressing that one word can be. We try to encourage the participants by explaining how *not* to respond (e.g., despair!) and what *to do*: first, recognize that the reviewers’ comments, however brutal, are a gift of that person’s time and expertise; then, read carefully and respond to each point; resubmit, if possible, with a gracious letter; or make the suggested changes and submit the manuscript elsewhere.

## Results

The workshop has been given in a variety of venues and has been adapted to cover time constraints as short as 1.5 hours to as long as 3 hours, demonstrating its flexibility. We have delivered this workshop to audiences composed of differing levels of learners including Executive Directors, medical trainees, faculty, and program administrators, and in local, national and international settings. In every instance, it has been lauded for its educational value and participants have rated the overall quality of the workshop, as well as individual components, as good or excellent and lessons learned as very useful and most useful (all above 4 on 1-5 scale). Narrative comments have indicated that participants found the overall organization, the sessions on structure and on multiple authors, and the workbook to be the most helpful aspects ( Table 1). One participant particularly appreciated the deconstruction of the content and skills practice, and wrote, “[The workshop] gave a broad picture, then broke down into feasible steps; makes process approachable.” Some participants identified the extensive practical experience of the faculty as the useful part of the workshop. Although the feedback from participants of the shorter sessions has been similar, they invariably suggested that a longer session would have been more useful. Of critical importance to us is that it has been found so valuable to participants at different venues that they have invited us to bring the workshop to their institutions and/or have recommended the workshop to others, demonstrating its educational value.

**Figure:**
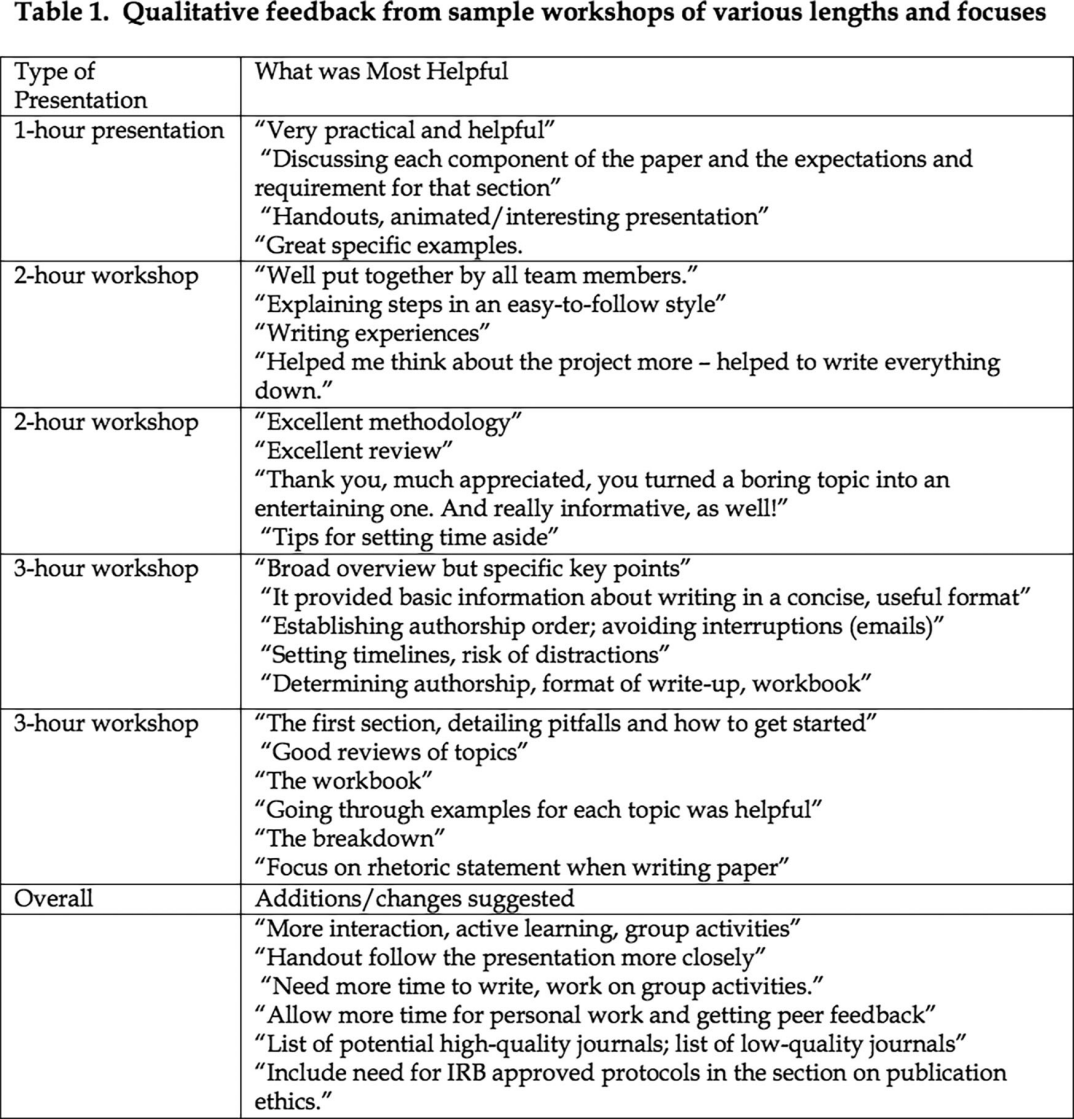


## Discussion

The content of this workshop is based on a didactic course on medical writing taught for more than 25 years that was restructured to incorporate pedagogy, psychosocial, and communication theories, using the complementary expertise of individuals from three disciplines. Guided by Kolb’s experiential learning, the didactic lecture was transformed into a highly interactive workshop. For example, the discussion on authorship described in Figure 1 has generated considerable discussion among the participants, as most of them have previously encountered such a dilemma and appreciate practical guidance. Over time, we have added and refined parts of the workshop according to ongoing needs assessments, feedback, and emerging information. For instance, in the section on Boundaries, we recently did some research on the toll that interruptions take on our time and our ability to concentrate. We explain how these interruptions affect one’s writing ability. To better explain the rhetorical component, we added a short explanation of Kinneavy’s theory of aims of discourse. Because of the increasing concern about the violation of ethical issues, we have sought to put more emphasis on this area to warn and protect the participants, as the repercussions can be life-changing.

Based on the participants’ feedback and the presenters’ own reflections, we adjusted some of the interactive components and eliminated one (writing their results), which we realized was impractical in most settings, although it could be implemented in a full-day writing retreat. We have offered various open-ended question/answer sessions and adjusted the sessions to allow for time to engage the participants in communicating with each other and with the presenters. The structure of the workshop is exceptionally flexible and can be replicated and adjusted as needed. For instance, we converted a one-hour presentation into a three-hour workshop by adding individual writing periods and several peer discussion times. In response to feedback, we also added a consultation component at the end, during which the presenters work with individuals and groups. Any of these components can be reduced, extended, or eliminated, depending on time constraints. Furthermore, the various topics can receive more or less attention, depending upon the audience’s particular needs.

We continue to keep the workshop dynamic by making accommodations in each session, based on feedback from participants. We realize that some clinician-educators will need more in-depth presentations and more structured time, so we continue to assess how to expand this workshop into half-day or full-day workshops or weekend retreats.

## Conclusion

Clinician-educators, like other academicians, often have little training in writing and need encouragement to launch a manuscript. This workshop offers both a practical means for engaging the writer and a wall of protection against possible pitfalls. It is easily adapted for use at different institutions.

## Take Home Messages

• This workshop is innovative and unique in its design, which incorporates educational, rhetorical, and social science theories into a didactic presentation on writing for the clinician-educator.
• By incorporating Kolb’s theory, we provide interactive opportunities for participants to engage one another and the presenters in discussions of various topics.
• We present “pitfall” to publishing, focusing particularly on ethics of publication, working with co-authors, and setting boundaries for one’s writing.
• We explain briefly Kinneavy’s theory of discourse and emphasize the relevance and necessity of crafting a rhetorical statement that succinctly states the value of the scholarly work and its place in medical education.
• We interweave didactic presentation with hands-on writing opportunities so the participants leave the workshop with a “skeleton” manuscript, ready to be completed for submission.

## Notes On Contributors

B. LEE LIGON, PHD, MA, MAR, is department medical writer, editor, and educator, and a core faculty member of the Center for Research, Innovation, and Scholarship in Medical Education, Department of Pediatrics, Baylor College of Medicine (BCM). She taught English literature and writing for more than 25 years at a private university, where she also created the Professional Writing Program.

RACHELLE WEINSTEIN, MSW, was part of the core faculty of the Center for Research, Innovation, and Scholarship in Medical Education, Department of Pediatrics, Baylor College of Medicine (BCM).

SATID THAMMASITBOON, MD, MHPE, is Associate Professor of Pediatrics, Critical Care at Baylor College of Medicine/Texas Children’s Hospital, and the Associate Director for the Center for Research, Innovation and Scholarship in Medical Education, Department of Pediatrics, Baylor College of Medicine.
