# Supplementary material for: Developing a Writing Workshop for Clinician-Educators: A Synergistic Integration of Ethics, Rhetoric and Education Theories, and Social Science
Source: MedEdPublish (2016). 2017 Jul 31;6:137. [Version 1] doi: 10.15694/mep.2017.000137 (PMC10885283; doi:10.15694/mep.2017.000137)
Supplement: Peer review history

# Reviewer report: Reviewer response for version 1

**DOI:** 10.21956/mep.19323.r28207

**Published:** 2017-09-02

**Authors:** Anthony David.M

**Affiliations:** 1 Soho

This review has been migrated. The reviewer awarded 4 stars out of 5 An excellent read! It is a well presented and lucid, 'do-able' paper which can be replicated. The adaptation that the program is undergoing makes it very useful. Being responsive to feedback from the participants and finetuning the program adds to the value of the workshop.

Reviewer Expertise:

NA

No decision status is available

# Reviewer report: Reviewer response for version 1

**DOI:** 10.21956/mep.19323.r28206

**Published:** 2017-08-18

**Authors:** Susan Van Schalkwyk

**Affiliations:** 1 Stellenbosch University

This review has been migrated. The reviewer awarded 3 stars out of 5 This is an interesting article that reminds us of the value of revisiting entrenched ways of doing (in this case the ‘didactic model’) and looking to innovative approaches to develop new models of engagement. The authors describe the ‘new look’ workshop series and emphasise how it is informed by existing theories that I thought were both relevant and accessible. The article is well-written and provides a solid overview of the approach that has now been adopted which will be useful for others wishing to amend or augment offerings of this nature. Some critical review/acknowledgement of the context within which academic writing and writing for publication occurs in many health professions education contexts and the factors (including external factors) that enable or constrain these processes would further strengthen this work. I also agree with other reviewers that the metaphor of football may not be familiar to all readers.

Reviewer Expertise:

NA

No decision status is available

# Reviewer report: Reviewer response for version 1

**DOI:** 10.21956/mep.19323.r28208

**Published:** 2017-08-04

**Authors:** Julie Hunt

**Affiliations:** 1 Lincoln Memorial University

This review has been migrated. The reviewer awarded 4 stars out of 5 This study describes and addresses some challenges or pitfalls in academic writing. It points to experiential learning theories, which was helpful. It may be most useful as an outline for educators who are trying to design a similar intervention to introduce at their institution. I agree with a previous reviewer that I would like to have seen higher-level data, perhaps quantifying how many grants or papers the participants had produced over a period of time. Perhaps the authors could look at that in a follow-up study.

Reviewer Expertise:

NA

No decision status is available

# Reviewer report: Reviewer response for version 1

**DOI:** 10.21956/mep.19323.r28209

**Published:** 2017-08-02

**Authors:** John Dent

**Affiliations:** 1 AMEE

This review has been migrated. The reviewer awarded 4 stars out of 5 I enjoyed reading this clearly-presented and informative paper. The Writing Workshop for clinician educators as described has been successfully used and developed. The application of Kolb’s cycle and Kinneavy’s communication triangle provide an informed approach to the topic.The paper would make a good paper in the 12 Tips series of Medical Teacher but, before that, may well benefit from the comments of a wider readership some of which have already been supplied by previous reviewers.

Reviewer Expertise:

NA

No decision status is available

# Reviewer report: Reviewer response for version 1

**DOI:** 10.21956/mep.19323.r28205

**Published:** 2017-07-31

**Authors:** Subha Ramani

**Affiliations:** 1 Harvard Medical School, Brigham and Women's Hospital

This review has been migrated. The reviewer awarded 5 stars out of 5 I thoroughly enjoyed reading this paper. It is clear that the authors are expert in staff / faculty development in the area of educational writing. They cover obvious challenges to writing and include some nuances that writers may forget. The paper is organized very intuitively so that educators can follow a sequence and a framework as they start writing. Ethics are covered, importantly criteria for authorship are clearly described. Lastly this paper provides recommendations on how to respond to reviewer suggestions and what not to do when manuscripts are rejected or revisions are recommended. All the recommendations are backed by theoretical constructs.The paper will be of interest to all health professions educators who are interested in writing and publishing. I have 2 constructive comments:- The authors mention baseball analogy, but later talk about football players. It is worth remembering that baseball is not played in many countries around the world and a 'home run' might be puzzling to some educators outside North America. Football, and I mean soccer, is much more universal and 'goal' is understood by most rather than 'homerun'.- While the bibliography is fairly extensive, I would suggest that educators also read Dr. Lorelei Lingard's articles on writing, in addition to this paper and the references..

Reviewer Expertise:

NA

No decision status is available

# Reviewer report: Reviewer response for version 1

**DOI:** 10.21956/mep.19323.r28210

**Published:** 2017-07-31

**Authors:** Trevor Gibbs

**Affiliations:** 1 AMEE

This review has been migrated. The reviewer awarded 3 stars out of 5 An interesting and well written paper that clearly outlines the pros, cons, pitfalls and solutions to effective writing, all described very succinctly as a course of variable length. Although well written I am not sure that this paper produces anything new, over and above the multiple papers that describe similar courses.I was quite disappointed to find that the authors relied so heavily upon a low level of evaluation- shouldn't we be looking more at the effect and output achieved from these courses?

Reviewer Expertise:

NA

No decision status is available
